# Stable topological insulators achieved using high energy electron beams

**DOI:** 10.1038/ncomms10957

**Published:** 2016-03-10

**Authors:** Lukas Zhao, Marcin Konczykowski, Haiming Deng, Inna Korzhovska, Milan Begliarbekov, Zhiyi Chen, Evangelos Papalazarou, Marino Marsi, Luca Perfetti, Andrzej Hruban, Agnieszka Wołoś, Lia Krusin-Elbaum

**Affiliations:** 1 Department of Physics, The City College of New York, CUNY, New York, New York 10031, USA; 2 Laboratoire des Solides Irradiés, École Polytechnique, CNRS, CEA, Université Paris-Saclay, 91128 Palaiseau cedex, France; 3 Laboratoire de Physique des Solides, CNRS, Université Paris-Saclay, Université Paris-Sud, 91405 Orsay, France; 4 Institute of Electronic Materials Technology, 01-919 Warsaw, Poland; 5 Institute of Physics, Polish Academy of Sciences, 02-668 Warsaw, Poland; 6 Faculty of Physics, University of Warsaw, 00-681 Warsaw, Poland

## Abstract

Topological insulators are potentially transformative quantum solids with metallic surface states which have Dirac band structure and are immune to disorder. Ubiquitous charged bulk defects, however, pull the Fermi energy into the bulk bands, denying access to surface charge transport. Here we demonstrate that irradiation with swift (∼2.5 MeV energy) electron beams allows to compensate these defects, bring the Fermi level back into the bulk gap and reach the charge neutrality point (CNP). Controlling the beam fluence, we tune bulk conductivity from *p*- (hole-like) to *n*-type (electron-like), crossing the Dirac point and back, while preserving the Dirac energy dispersion. The CNP conductance has a two-dimensional character on the order of ten conductance quanta and reveals, both in Bi_2_Te_3_ and Bi_2_Se_3_, the presence of only two quantum channels corresponding to two topological surfaces. The intrinsic quantum transport of the topological states is accessible disregarding the bulk size.

## Abstract (web summary)

Defects in solids may introduce additional charges that influence the overall charge transport behaviour. Here, Zhao *et al*. use swift electron beams to compensate charge defects, which effectively tune Bi_2_Te_3_ and Bi_2_Se_3_ from *p*-type to *n*-type while preserving their topological properties.

## Graphical abstract

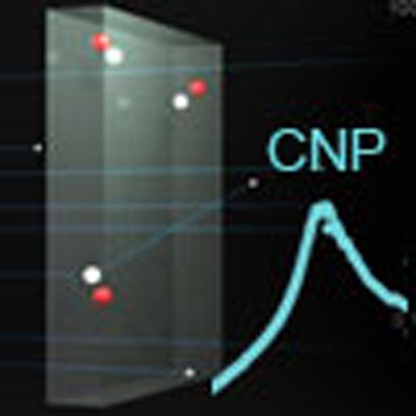

Unconventional quantum matter can be easily hidden within the rich existing library of condensed matter and to recognize it often novel concepts have to be invoked. It also takes a profound understanding of the real material constraints that can prevent the unconventional properties from being detected. Three-dimensional (3D) topological insulators are a spectacular example of this^1^—narrow-band semiconductors, well known for their high performance as thermoelectrics^2^, they were discovered to support unusual gapless robust two-dimensional (2D) surface states that are fully spin-polarized with Dirac-type linear electronic energy-momentum dispersion^3^,^4^, which makes them protected against backscattering by disorder^3^,^4^,^5^,^6^. These materials have narrow bulk gaps (∼200–300 meV) and have charge carriers donated by intrinsic crystalline lattice defects such as vacancies and antisites^7^. As a result, the conduction through the bulk and its intermixing with surface channels is what largely denies direct access to surface charge transport required for the implementation in spin-based nanoelectronics^8^ or fault-tolerant topological quantum computing^4^.

Extensive attempts to reduce the contribution of bulk carriers, involving techniques such as nanostructured synthesis/growth^9^,^10^, chemical doping^11^ or compositional tuning^12^, have relied on electrostatic gating of micro- or nanostructures comprising tens of nanometre thin films^13^ or similarly thin exfoliated crystals^14^ to gain less ambiguous access to the surface states. Here we demonstrate that bulk conductivity in topological insulators (TIs) can be decreased by orders of magnitude to charge neutrality point on a large (depth) scale by the controlled use of electron beams, which for energies below ∼3 MeV are known to produce a well-defined, stable and uniform spread of Frenkel (vacancy-interstitial) pairs^15^ within their penetration range of hundreds of micrometres. The combined effect of these pairs is to compensate for the intrinsic charged defects responsible for the conductivity of the bulk while crystal lattice integrity is maintained and, as we demonstrate by angularly resolved photoemission spectroscopy (ARPES), the topological Dirac surface states remain robust. Stable surface conduction channels are achieved when a sufficient irradiation dose is followed by the optimally engineered annealing protocol, thereby resolving one of the key limitations of bulk TIs.

## Results

### Compensating bulk carriers using electron beams

**Figure 1:**
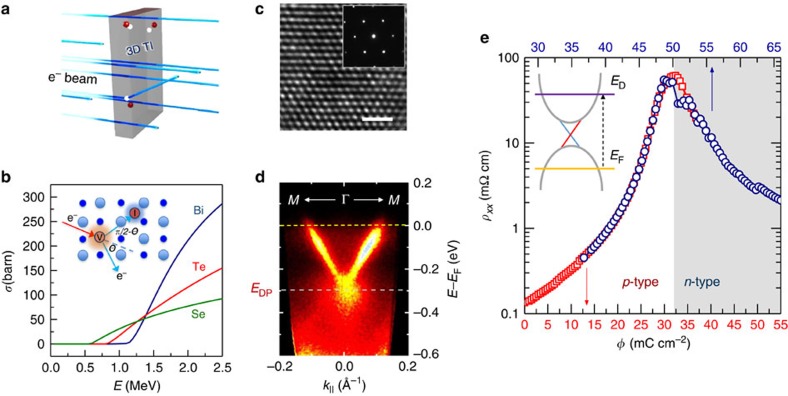
Tuning bulk conductivity of topological materials by swift particle irradiation (**a**) Energetic electron beams can penetrate solids to a depth of many tens of micrometres. Electron irradiation affects the bulk but not the robust topological surfaces. (**b**) Impinging electrons induce formation of Frenkel vacancy-interstitial pairs (inset), which act to compensate the intrinsic bulk defects. Main panel: calculated crosssections *σ* for Frenkel pair production in Bi, Te and Se sublattices as a function of electron energy *E*, assuming displacement energy^16^ ∼25 eV. Energy thresholds, *E*_th_, in *σ* are set by atomic weight; choosing 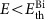 or 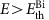 allows to tune Fermi level in both *p-* and *n-*type TIs. (**c**) Transmission electron microscopy image of Bi_2_Te_3_ with electron dose *ϕ*=1 C cm^−2^; the atomic displacements of ∼1 per 5,000 are not seen. Scale bar, 1 nm. (**d**) ARPES spectrum taken at 130 K (see ‘Methods' section) of a Bi_2_Te_3_ crystal after irradiation with high electron dose of 1.7 C cm^−2^. The irradiated sample becomes *n*-type, with Dirac point at *E*_DP_=−290(10) meV relative to the Fermi level *E*_F_ (just below the bottom of the bulk conduction band). It demonstrates electronic robustness of Dirac spectrum against electron irradiation. (**e**) Resistivity of *p-*type Bi_2_Te_3_ irradiated with 2.5 MeV electrons versus dose *ϕ* (red squares) measured *in situ* at 20 K shows about three orders of magnitude increase at the charge neutrality point (CNP) where the conduction is converted from *p-* to *n-*type, moving the Fermi level *E*_F_ across the Dirac point (see cartoon). Cycling to room temperature reverses the process, which can be recovered by further irradiation (blue circles) and stabilized. The conversion from *n-* to *p-*type is obtained in a material such as Pb-based TI, where vacancies on the Pb sites are known to be of acceptor type.

Under exposure to light particles such as electrons, the production of vacancy-interstitial point defects^16^ in solids known as Frenkel pairs is well established. The pair formation has an energy threshold which scales with atomic weight—it is high for heavy Bi and is lower for lighter Te and Se, and the effective cross-section *σ* for pair creation on different sublattices in materials containing these elements depends on the projectile energy (Fig. 1a,b and Supplementary Figs 1,2). This defines a pair-production window: below these thresholds, the given sublattice becomes immune to irradiation, whereas at energies above ∼3 MeV, clustering and even nuclear reactions^17^ may occur. The process of Frenkel pair formation is straightforward and can be well controlled. The charge is primarily delivered by introduced vacancies, since at room temperature interstitials do not contribute owing to their much lower (compared with vacancies) migration barriers. For electron beam energies above ∼1.5 MeV, the effective cross-section *σ* on a Bi sublattice is the highest and we will show that donor type defects on this sublattice do prevail. For energies below Bi threshold (∼1.2 MeV), the creation of Frenkel pairs will be mostly on Se or Te sublattices and, correspondingly, by tuning electron beam energy a different mix of donor/acceptor defects and a net acceptor charge doping can be expected.

Generally, crystal growth of tetradymite crystals such as Bi_2_Te_3_ and Bi_2_Se_3_ results in equilibrium defect configurations comprising vacancies and antisites on both sublattices^7^,^18^. The net free charge balance that delivers carriers to bulk conduction or valence bands from these defects can be varied by growth conditions or doping^11^. In undoped Bi_2_Se_3_, where Se vacancies are presumed to dominate, the net conduction is by electron carriers or *n*-type. In Bi_2_Te_3_, where antisites are prevalent^19^ the conductivity is usually *p*-type, namely by hole carriers, although by varying stoichiometry it has been grown of either conductivity type. Without a priori knowledge of the net donor or acceptor flavour of the pairs on different sublattices, we have chosen first to irradiate *p*-type topological materials Bi_2_Te_3_ and Ca-doped^11^ Bi_2_Se_3_ with 2.5 MeV electron beams that create Frenkel pairs on all sublattices according to *σ*(*E*), shown in Fig. 1b and Supplementary Fig. 2. Our results demonstrate that in the above-mentioned Bi-based TIs at this energy, the net flavour is of donor type. It is also important to note that even for relatively high (≳1 C cm^−2^) electron irradiation doses, the lattice parameters remain unchanged (see Supplementary Fig. 3).

### Ambipolar conduction tuned by irradiation dose

**Figure 2:**
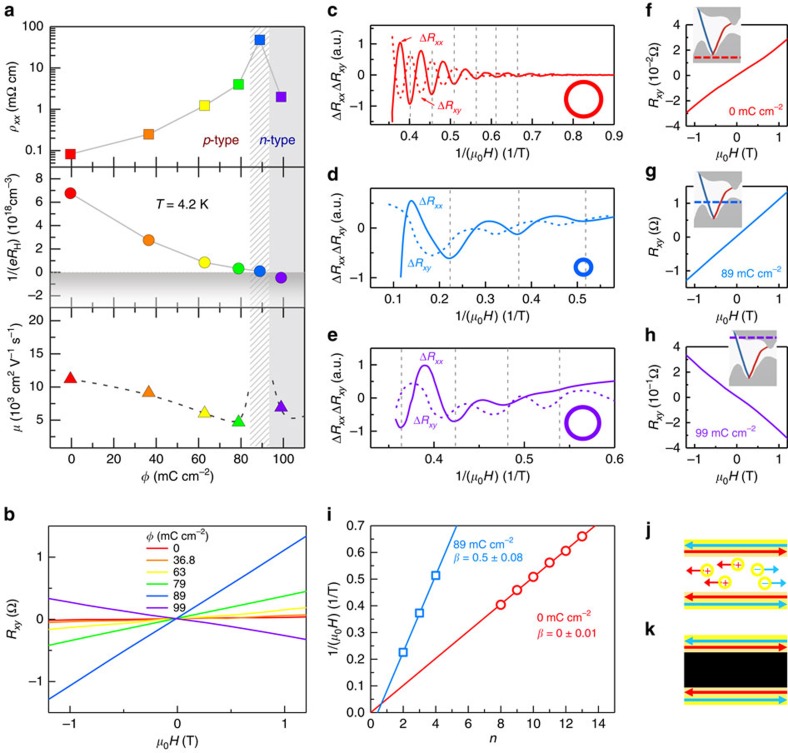
Transport and quantum oscillations across charge neutrality point. (**a**) Longitudinal resistivity *ρ*_*xx*_ of Bi_2_Te_3_ crystals irradiated to different terminal doses and measured at 4.2 K shows three orders of magnitude increase at CNP where the bulk is converted from *p-* to *n-*type, as indicated by the sign change of the slope *dR*_*xy*_/*dH* of Hall resistance *R*_*xy*_ shown in **b**. Inverse Hall coefficient 1/*eR*_H_ ≃−*n*_b_ gives an estimate of net carrier density *n*_b_ as a function of dose. At CNP *R*_H_ changes sign. Mobility just above CNP appears higher than just below, with the expected trend of *μ* at CNP indicated by dash. Outside the immediate vicinity of CNP, effective carrier mobilities *μ* estimated from 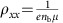 in the Drude model are not much affected by the irradiation process, confirming that scattering events by Frenkel-pair point defects are scarce and the main effect is charge compensation. Shubnikov-de Haas (SdH) quantum oscillations of Δ*R*_*xx*_ and Δ*R*_*xy*_ in Bi_2_Te_3_ with magnetic field applied along the *c* axis: (**c**) before *e*-beam exposure, (**d**) irradiated to a dose 89 mC cm^−2^ and (**e**) to a dose 99 mC cm^−2^. The corresponding sizes of Fermi surfaces obtained from SdH (Supplementary Table 1) are cartooned as circles. (**f**–**h**) Low-field *R*_*xy*_ for the samples in (**c**–**e**). The Fermi level (dashed line) is moving from the bulk valence band (BVB) in **f** to the bulk conduction band (BCB) in **h** as illustrated in outsets by the cartoons of calculated^5^ Bi_2_Te_3_ band structure. (**i**) The Landau level index plot versus field minima in the SdH oscillations in **c** (red) and **d** (blue) yields an estimate of Berry phase 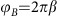, see text. (**j**,**k**) Cartoons of a TI with conducting bulk before irradiation (**j**) and after, with ideally only topological surfaces conducting (**k**).

Longitudinal resistivity *ρ*_*xx*_ of the initially *p-*type Bi_2_Te_3_ measured at 20 K *in situ* in the irradiation chamber (see ‘Methods' section) as a function of irradiation dose is shown in Fig. 1e. Most immediately notable features in the figure are a nearly three orders of magnitude resistivity increase to a maximum 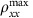 and the observed ambipolar conduction as a function of irradiation dose with well-distinguished *p* (hole) and *n* (electron) conduction regions. The resistivity maximum 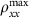 (the conductivity minimum 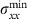) is at the charge neutrality point where conduction is converted from *p-* to *n-*type, as determined from Hall resistivity (see Fig. 2). The same type conversion is observed in Ca-doped Bi_2_Se_3_ (Supplementary Fig. 4a and Supplementary Note 1), which is also *p-*type^11^. As long as the terminal irradiation dose *ϕ* is relatively low, below ∼0.1 C cm^−2^, *ρ*_xx_(*ϕ*) traces its shape upon temperature cycling to room temperature and back to 20 K, with 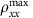 reproduced by the next irradiation cycle. Here it is apparent that the value of *ϕ*_max_ at the charge neutrality point is not universal; it depends on the starting free carrier concentration *n*_b_ but can be straightforwardly scaled using universal slope 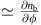 of quasi-linear variation of *n*_b_ versus dose (Fig. 2a and Supplementary Figs 4b and 5).

### Longitudinal and Hall resistivities across CNP

*Ex situ* measured resistivities of Bi_2_Te_3_ crystals irradiated to different terminal doses and taken to room temperature before the chill-down to 4.2 K are in full correspondence with the *in situ* results. Figure 2a shows three orders of magnitude increase in *ρ*_*xx*_ to the maximum, 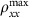, at *ϕ*_max_≈90 mC cm^−2^ higher than the *in situ ϕ*_max_ (Fig. 1e) likely owing in part to some defect migration (mostly interstitials^16^) above 100 K. The *p-* to *n-*type conversion is clearly seen in Hall resistance *R*_*xy*_ (Fig. 2b,f–h) flipping its slope d*R*_*xy*_/d*H* and Hall coefficient 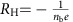 changing sign in the conversion region. Near CNP, the net residual bulk carrier density *n*_b_=*n*_D_−*n*_A_ is very low (*n*_D_ and *n*_A_ are concentrations of donors and acceptors, respectively). In this region, local charge fluctuations can be very large creating inhomogeneity akin to a network of puddle-like *p*–*n* junctions that nonlinearly screen random potential on long length scales^20^ and a simple estimate of *n*_b_ is no longer appropriate^14^. Mobilities in our crystals are significantly higher (*μ*≅7,000–11,000 cm^2^ *V*^−1^ s^−1^, see Supplementary Table 1) than a commonly observed ≲1,000 cm^2^ V^−1^ s^−1^ range^14^ near the CNP region.

### Shubnikov–de Haas oscillations and Berry phase

The change in net carrier density induced by irradiation is reflected in the observed Shubnikov–de Haas (SdH) quantum oscillations (Fig. 2c–e) of sheet and Hall resistances, Δ*R*_*xx*_ and Δ*R*_*xy*_, from which an estimate of the Fermi surface size can be obtained (Supplementary Table 1). The Fermi vector in pristine crystals *k*_F_≈0.025 Å^−1^ is consistently larger than after irradiation and, for example, the dose *ϕ*≅89 mC cm^−2^ that tunes the crystal close to CNP results in *k*_F_≈0.014 Å^−1^. The corresponding net carrier densities are *n*_b_=5.06 × 10^17^ cm^−3^ and *n*_b_≃1.08 × 10^17^ cm^−3^, respectively. At higher irradiation dose *ϕ*≅99 mC cm^−2^, with *k*_F_≈0.022 Å^−1^ Fermi surface size becomes comparable to the pristine material. A comparison of these numbers with carrier densities obtained independently from Hall data (Supplementary Table 1) reveals a good agreement between the two techniques near CNP but about an order of magnitude higher estimate of *n*_b_ by the latter^21^ well outside the CNP region. Berry phase 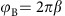 can be estimated from SdH oscillations using a semiclassical description^22^ 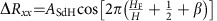, where *A*_SdH_ is the oscillation amplitude, *H*_F_ is the frequency in 1/*H* and *β* is the Berry factor. Near CNP, the obtained Berry factor is *β*=0.5±0.06 as expected for the topological Dirac particles, while outside the CNP region, a trivial value of *β*≃0 is obtained (Fig. 2i).

### Nonequilibrium defect compensation at low irradiation dose

**Figure 3:**
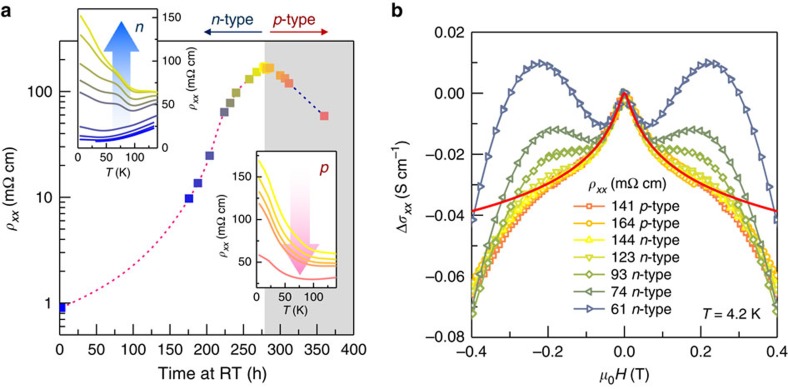
Time evolution of charge transport across charge neutrality point in Bi_2_Te_3_ after low-dose exposure. (**a**) Evolution of longitudinal resistivity *ρ*_*xx*_ measured at 4.2 K after cycling to room temperature (RT) for a crystal irradiated with dose *ϕ*=90 mC cm^−2^. Each RT dwell time is coded with a different colour. Resistivity is seen to cross charge neutrality point (CNP) in reverse from *n-*type back to *p*-type. It is consistent with slow migration (hundreds of hours at RT) of vacancies (in accord with ∼0.8 *eV* migration barriers, see text) and shows that CNP can be reached by designing a suitable thermal protocol. Insets show *ρ*_*xx*_(*T*) for *n-*type region (upper left) and *p-*type region (lower right). (**b**) Change in magnetoconductance (MC) at different RT dwell times; here MC evolves from a quadratic field dependence of a typical bulk metal at short RT dwell times, through a complex region dominated by the charge-inhomogeneous bulk, to a weak antilocalization (WAL) region showing the characteristic low-field cusp near CNP. Here the data were normalized to the value at zero field. A fit to 2D localization theory^26^ is shown as red line, see text.

The stability of net carrier density crucially depends on the terminal irradiation dose. First, we illustrate that for low terminal electron doses (*ϕ*≲0.1 C cm^−2^), the resistivity is not stable as it evolves from the *n*-region after irradiation through CNP back into the *p*-region. The experiment was to simply change the dwelling time at room temperature to allow for vacancies to diffuse. The result is the reverse conversion (Fig. 3a) from metallic-like resistivity on the *n*-side just after irradiation, through insulating resistivity at CNP (after nearly 250 h at room temperature) and back to a weakly semiconducting-like resistivity on the *p*-side. As the system evolves across CNP, a weak antilocalization quantum interference correction^23^ to classical magnetoresistance (MR) emerges in the non-equilibrated conversion region (Fig. 3b), with a complex MR structure and nonlinear Hall resistivity (see Supplementary Fig. 6), likely reflecting a presence of two carrier types. From isochronal annealing experiments (Supplementary Fig. 7 and Supplementary Note 2), we estimate energy barriers controlling defect migration to be ∼0.8 eV corresponding to ∼9,000 K, in line with literature values for vacancy migration in solids^16^. We conclude that at low terminal doses while defect migration is sluggish, the equilibrium is not attained.

### Achieving stability at CNP with high irradiation dose

**Figure 4:**
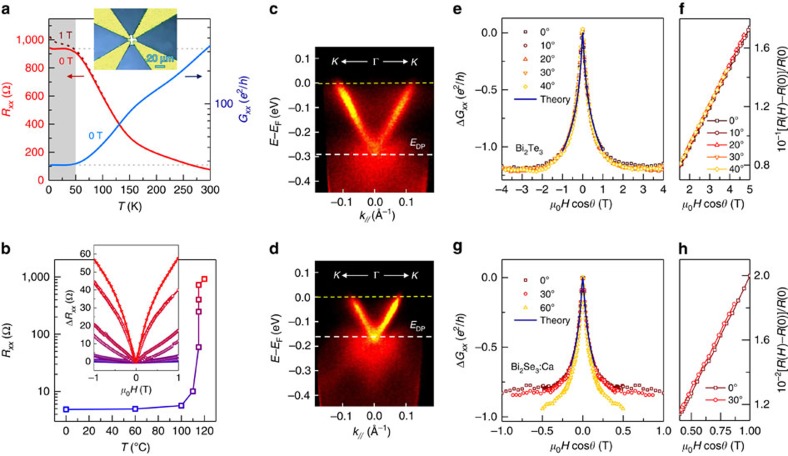
Stable charge neutrality point and 2D conductance in e-beam irradiated topological materials. (**a**) Sheet resistance *R*_*xx*_ versus temperature of Bi_2_Te_3_ crystal at the charge neutrality point (red line) exhibits a plateau at low temperatures—a thumbprint of 2D surface conduction, see right panels in **e** and **f**). We note that in Bi_2_Te_3_, CNP and Dirac point do not coincide, with the Dirac point situated within a valley in the bulk valence bands^5^, and 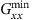 is expected to reflect that^23^. Magnetic field breaks time reversal symmetry and gaps Dirac bands, resulting in localizing behaviour shown as dash. Inset: optical image of the crystal showing van der Pauw contact configuration used. (**b**) Annealing protocol with time steps Δ*t*=30 min implemented to tune Bi_2_Te_3_ crystal with dose 1 C cm^−2^ back to stable CNP. Inset: magnetoresistance at 1.9 K after each annealing step, with colours matched to indicate different annealing temperatures. (**c**,**d**) ARPES spectra of a Bi_2_Te_3_ crystal irradiated with electron dose of 1.7 C cm^−2^ taken along Γ−*K* direction in the Brillouin zone. (**c**) Before annealing, the irradiated sample is *n*-type, with Dirac point at *E*_DP_∼−290(10) meV relative to the Fermi level *E*_F_. (**d**) After annealing at 120 °C, Dirac point upshifts to a binding energy of *E*_DP_∼−160(10) meV. The same shift is seen for the scans along Γ−*M*. (**e**) WAL low-field quantum interference correction to the linear-in-field magnetotransport ((**f**), also see text) at CNP in a Bi_2_Te_3_ crystal at 1.9 K with its characteristic low-field cusp. The 2D character of WAL is evident in its scaling with transverse field *H*_‖_=*H*cos*θ*, where *θ* is the tilt angle of the field measured from sample's *c* axis. A fit to 2D localization (HLN) theory^26^ (solid line) confirms that the contribution is only from two surfaces and yields a dephasing field 
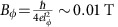
. (**f**) Linear magnetoresistance at CNP shows 2D scaling with *H*_‖_. (**g**) WAL contribution at CNP in a Bi_2_Se_3_:Ca(0.09%) crystal at 1.9 K also scales with *H*_‖_. At high fields outside the cusp, the scaling is seen to fail for *θ*≳60°. A fit to HLN theory (solid line) again confirms the contribution only from two surfaces and yields a smaller dephasing field *B*_*ϕ*_∼0.004 T (corresponding to dephasing length *l*_*ϕ*_∼220 nm). (**h**) Linear magnetoresistance at CNP also scales with *H*_‖_.

Next we demonstrate that stability can be controlled and optimized in a reverse conversion process by the annealing protocol when the terminal electron dose is sufficiently high. This is the key part of the material modification process by electron irradiation; without it surface transport via electron irradiation could not be obtained^24^. We demonstrate this for the samples exposed to the dose 10 times higher, *ϕ*=1 C cm^−2^. Figure 4a shows temperature dependence of sheet resistance *R*_*xx*_ and conductance *G*_*xx*_ of Bi_2_Te_3_ tuned back to CNP through a thermal protocol shown in Fig. 4b, where it remained for months of testing (Supplementary Figs 8,9 and Supplementary Note 3). As clearly seen in ARPES (Fig. 4c,d and Supplementary Fig. 10), under this annealing protocol Dirac dispersion remains preserved. The effect of tuning conduction from the irradiation-induced *n*-type (Fig. 4c) back towards CNP (Fig. 4d) is a relative shift of the Fermi level toward the Dirac point, consistent with transport data and with the calculated band structure^5^ of Bi_2_Te_3_ (see cartoons in Fig. 2f–h).

*R*_*xx*_(*T*) increases exponentially as the temperature decreases, turning below ∼200 K into variable range hopping (Supplementary Fig. 11 and Supplementary Note 4). This bulk behaviour is cut off at low temperatures when the contribution from the surface becomes comparable and a temperature-independent surface transport with minimum conductance^14^ 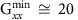
*e*^2^/*h* reveals its quantum nature. The 2D character of this region is witnessed by MR that depends only on the transverse component of magnetic field *H*_‖_=*H*cos*θ* (Fig. 4e–h). Unlike the quadratic-in-field MR of a conventional metal, the large (≳10%) magnetoresistance at CNP (Fig. 4a) is found to be linear in field (Fig. 4f,h).

The topological protection of the surface states near CNP can be tested in at least three ways. One, by breaking time reversal symmetry (TRS) which should gap out Dirac states. Another, by the appearance of topological Berry phase^4^ of *π* (see Fig. 2i). And yet another, by detecting 2D weak antilocalization (WAL) of Dirac particles travelling through time-reversed paths^23^ associated with this Berry phase. Figure 4a shows that applying TRS-breaking magnetic field indeed causes *R*_*xx*_(*T*) at the lowest temperatures to upturn, showing localizing behaviour consistent with opening of the Dirac gap (see, for example, ref. 25). From this, we conclude that near CNP, even with the complex Bi_2_Te_3_ band structure, topological conduction channels dominate.

### Stable 2D quantum transport at CNP

As we approach CNP, WAL quantum correction to classical conductivity emerges at low magnetic fields as a characteristic positive magnetoresistance cusp^23^ (inset in Fig. 4b). In close proximity to CNP, the corresponding cusp in negative magnetoconductance scales with the transverse field *H*_‖_=*H*cos*θ* (Fig. 4e,g), confirming its 2D character. In this case, the number *n*_Q_ of quantum conduction channels contributing to WAL can be estimated from 2D localization theory^26^


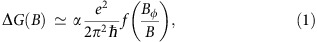


where Δ*G*(*B*) is the low-field quantum correction to 2D magnetoconductance, coefficient *α*=*n*_Q_/2 equals to 1/2 for a single 2D channel, 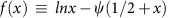, 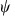 is the digamma function, and field 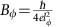 is related to the dephasing length *l*_*ϕ*_ of interfering electron paths. In Bi_2_Te_3_ at CNP, the fit (in Fig. 4e) yields *α*≃1.26±0.1 corresponding to *n*_Q_∼2, smaller than 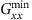. The obtained value of ∼2 is quite remarkable since ‘universality' of *n*_Q_ has been questioned^27^ given a likely formation of subsurface 2D electron gas states of bulk origin. In Bi_2_Se_3_, where the Dirac point is expected to coincide with CNP, the fit similarly yields *α*≃1.12±0.1 (Fig. 4g), corresponding to two 2D quantum channels we associate with two independent topological surfaces.

Finally, we remark that using thermal protocol illustrated in Fig. 4b, we found that once CNP is reached, the system remains there for months on cycling, which was the duration of our experiments. This robustness suggests that at higher electron doses, vacancies are likely correlated; for example, di-vacancies can form^16^,^28^ and it is known that point-defect complexes can be stabilized^29^ in systems with multiple sublattices. With the choice of electron beam energy and terminal electron dose controlling the stability of pairs, and with a suitably designed thermal tuning to charge neutrality, the high-energy electron irradiation offers a path to large scale access to topological states.

## Methods

### Crystal growth and structural characterization

The standard Bridgman-Stockbarger technique employing a vertical pull through the temperature gradient was used to grow single crystals of Bi_2_Te_3_ and Ca(0.09%)-doped Bi_2_Se_3_. X-ray diffraction of crystals was performed in a Panalytical diffractometer using Cu K*α* (*λ*=1.5405 Å) line from Philips high intensity ceramic sealed tube (3 kW) X-ray source with a Soller slit (0.04 rad) incident and diffracted beam optics. Transmission electron microscopy was performed at Evans Analytical Group.

### Angularly resolved photoemission spectroscopy

ARPES measurements were performed on the FemtoARPES setup^30^ using a Ti:sapphire laser operated at 0.25 MHz repetition rate. The specimens were probed with the fourth harmonic (at 6.26 eV), with an overall spectral resolution better than 30 meV. The data were taken along Γ*-M* and Γ*-K* directions. We found no substantial differences for measurements performed on several cleaved surfaces.

### Electron irradiation

Electron irradiations were carried out in NEC Pelletron-type electrostatic accelerator in Laboratoire de Physique des Solides at École Polytechnique, Palaiseau, configured with a low-temperature target maintained at ∼20 K in a chamber filled with liquid hydrogen fed from a close-cycle refrigerator. All irradiations were performed with samples kept at 20 K, below the mobility threshold of the interstitials which tend to be more mobile than vacancies^16^—this ensured the stability of all charges introduced by the irradiation process. It also allowed us to evaluate thermal migration of interstitials during the annealing process. The size of the 2.5 MeV electron beam spot was reduced to 5 mm by a circular diaphragm aperture, with uniformity of the beam current ensured with a constant beam sweep in *x* and *y*-directions at two non-commensurate frequencies. Careful calibration of the beam current density, typically of 2 μA on a 0.2 cm^2^ surface, was performed by periodic introduction of a Faraday cage between the diaphragm and the sample chamber and the measurement of current collected on the control metallic sample. Beam current densities, limited to 10 μC cm^−2^ s^−1^ by the cooling rate, allowed modifications of carrier concentration on the order of 10^20^ cm^−3^.

### Transport measurements

The system was set up for *in situ* transport measurements that could be monitored as a function of electron dose in real time. Crystals used in the irradiation experiments were typically 15 μm thick with spark-weld electrical contacts placed in van der Pauw contact configuration. *Ex situ* transport and Shubnikov–de Haas oscillations measurements were performed in Quantum Design Physical Property Measurement System equipped with 14 T magnet. In *ex situ* measurements, when samples were exfoliated to thicknesses of 200 nm and below, lithographically designed contacts with Ti/Au metallurgy were used (see Fig. 4a). Annealing experiments up to 200 °C (such as shown in Fig. 4b and Supplementary Note 2) were performed in a box furnace in flowing nitrogen.

## Additional information

**How to cite this article:** Zhao, L. *et al*. Stable topological insulators achieved using high energy electron beams. *Nat. Commun.* 7:10957 doi: 10.1038/ncomms10957 (2016).

## Supplementary Material

Supplementary Information Supplementary Figures 1-11, Supplementary Table 1, Supplementary Notes 1-4 and Supplementary References
