# Supplementary material for: Stable topological insulators achieved using high energy electron beams
Source: Nat Commun. 2016 Mar 10;7:10957. doi: 10.1038/ncomms10957 (PMC4792950; doi:10.1038/ncomms10957)
Supplement: Supplementary Information — Supplementary Figures 1-11, Supplementary Table 1, Supplementary Notes 1-4 and Supplementary References [file ncomms10957-s1.pdf]

Supplementary Figures

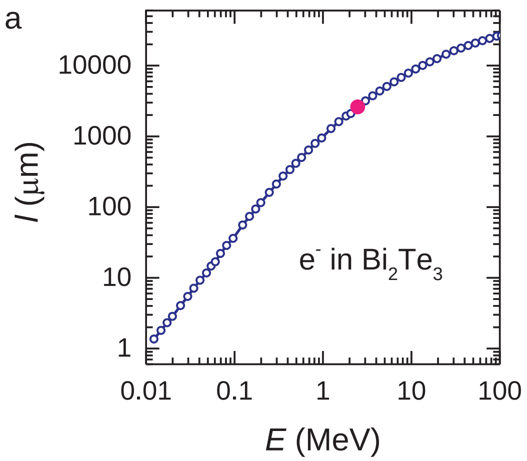

Supplementary Figure 1| Penetration range of electrons beams in Bi_2Te_3 and Bi_2Se_3. Penetration depth of electrons in (a) Bi_2Te_3 and (b) Bi_2Se_3 calculated using NIST ESTAR simulator (http://physics.nist.gov/PhysRefData/Star/Text/ESTAR.html). The penetration depth at 2.5 MeV (red dots) used in our experiments is $> 2,000$ μm and the resulting depth profile of vacancies is uniform over hundreds of microns.

Particle beams such as Ne^+ and He^{2+} (alpha particles) have a much shorter penetration depth than electrons and are known to produce defect cascades and extended defects. For example, 150 keV Ne^+ ion irradiation has a nonuniform depth profile and shows a pileup of defects at about 0.2μm. Such irradiations were shown to *increase* electron concentration away from the surface states and to stabilize Fermi level high up in the bulk conduction bands [1] of n-type Bi_2Te_3 and Bi_2Se_3. We note that charge doping by electron irradiation in band semiconductors such as PbTe has been explored in the eighties [2]. One previous attempt in Bi_2Te_3 [3] used low e-beam fluences with the target kept at room temperature. In this process, the irradiated samples displayed none of the features associated with surface carriers or even showed semiconducting like behavior of resistivity *vs.* temperature. In our experiments, irradiation were performed in liquid hydrogen. This allowed us to control pair formation during the energetic irradiation process and develop a scheme under which stable vacancy content that compensated native charged defects was established.

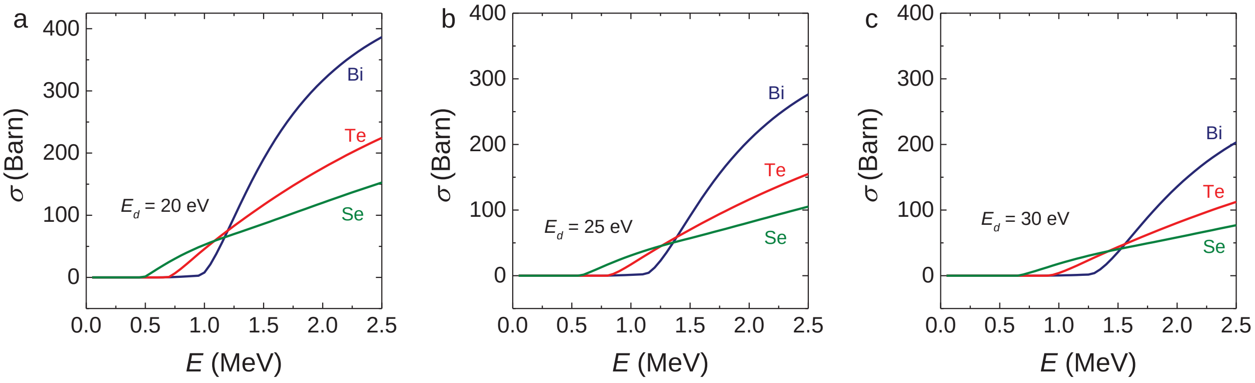

Supplementary Figure 2| Energy thresholds for the Frenkel pair creation on Bi, Te and Se sublattices. Calculated crosssections σ for Frenkel pair production on Bi, Te and Se sublattices as a function of electron energy E with the displacement energy E_{d} of (a) 20 eV, (b) 25 eV, and (c) 30 eV. Energy thresholds, E_{th}, in σ are not sensitive to E_{d} and are largely set by the atomic weight.

Empirical determination of displacement energies E_{d} is relatively straightforward in metals [4]. It is much more complex in semiconductors or semimetals where defects can affect both carrier concentration and mobility and there is an argument given by Seitz [5] that E_{d} can be related to sublimation energy. Damask-Dienes [4] give E_{d} for Bi, Te and Se in the range of 20 - 30 eV. We have performed calculations of Frenkel-pair production crosssections for all three sublattices in this range. The calculation is based on Rutherford collision of a relativistic electron with energy E with a nucleus of mass M, where the transferred energy $E = E_{\max}\sin^2(\theta)/2$ may range from zero in a glancing collision to a maximum energy $E_{\max} = 2E(E + 2mc^2)/Mc^2$ transferred in a head-on collision. The pair production crosssection is given by $\sigma = \frac{2\pi}{E_{\max}} \int_{E_{\mathrm{d}}}^{E_{\max}} \frac{\mathrm{d}\sigma(E)}{\mathrm{d}\theta} P_{\mathrm{d}}(E)\mathrm{d}E$, where the differential crosssection is a relativistic extension of classical Rutherford formula derived by Mott [6, 7] and $P_{\mathrm{d}}(E)$ is the probability the atom was pushed in the direction $\frac{\pi}{2} - \frac{\theta}{2}$ (see main Fig. 1b). Both the energy thresholds and the ratios of pair production on Bi and Te or Bi and Se sublattices are not very sensitive to E_{d}. For this reason we have chosen $E_{\mathrm{d}} = 25$ eV, in the midrange.

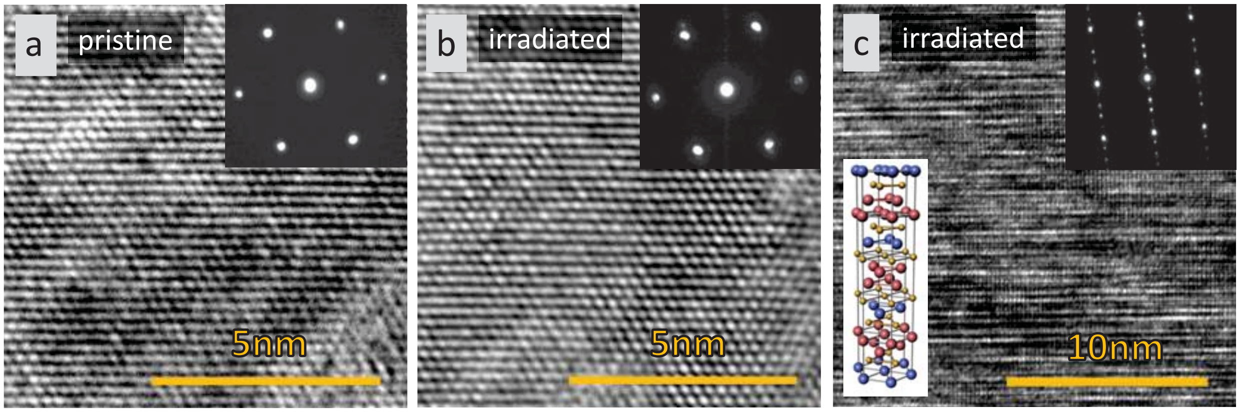

Supplementary Figure 3| Transmission Electron Microscopy of Bi_2Te_3 exposed to 2.5 MeV e-beams. TEM images and diffraction spots of (a) pristine and (b) irradiated Bi_2Te_3 show the same hexagonal lattice in the *ab*-plane. (c) Layered van der Walls structure along the *c*-axis (normal to $(00\bar{1})$ cleavage plane) after irradiation. Inset: Rhombohedral layered structure of Bi_2Te_3 constructed using lattice parameters ($a = 4.38\mathring{A}$ and $c = 30.45\mathring{A}$) from the X-ray diffraction (XRD). The van der Walls structure has three quantuple layers per unit cell [8].

Shown are transmission Electron Microscopy (TEM) images of Bi_2Te_3 irradiated to a dose $\phi = 1\text{C/cm}^2$. At this dose and after 40 hours at room temperature, we estimate the number of the atomic displacements as follows. For 2.5 MeV electrons the effective cross-section is $\sigma \sim 300$ barns (1 barn $= 1 \times 10^{-24}$ cm^2). With the dose of 0.1 C/ cm^2 $= 6.25 \times 10^{17}$ electrons/cm^2 we have 0.000187 d.p.a on Bi sublattice, corresponding to a roughly ~ 1 per 5,000 atoms ejected, which are not easily discerned in the image. The images of irradiated and pristine samples are indistinguishable.

We note that electron irradiation induced vacancy - interstitial (Frenkel) pairs [9, 10] can be well controlled by the integrated irradiation dose (beam fluence) to a very low carrier concentration level ($\lesssim 10^{13}$ cm^{-3}). Electron irradiation produces donor-type doping in both Bi_2Te_3 and Ca-doped Bi_2Se_3 compounds (with a slightly higher rate $\frac{\partial n_b}{\partial \phi}$ for Bi_2Se_3). At the present stage we cannot differentiate between the doping action of vacancies on Bi and Te/Se sites, however, we clearly demonstrate bulk charge compensation of *p*-type TIs across charge neutrality point.

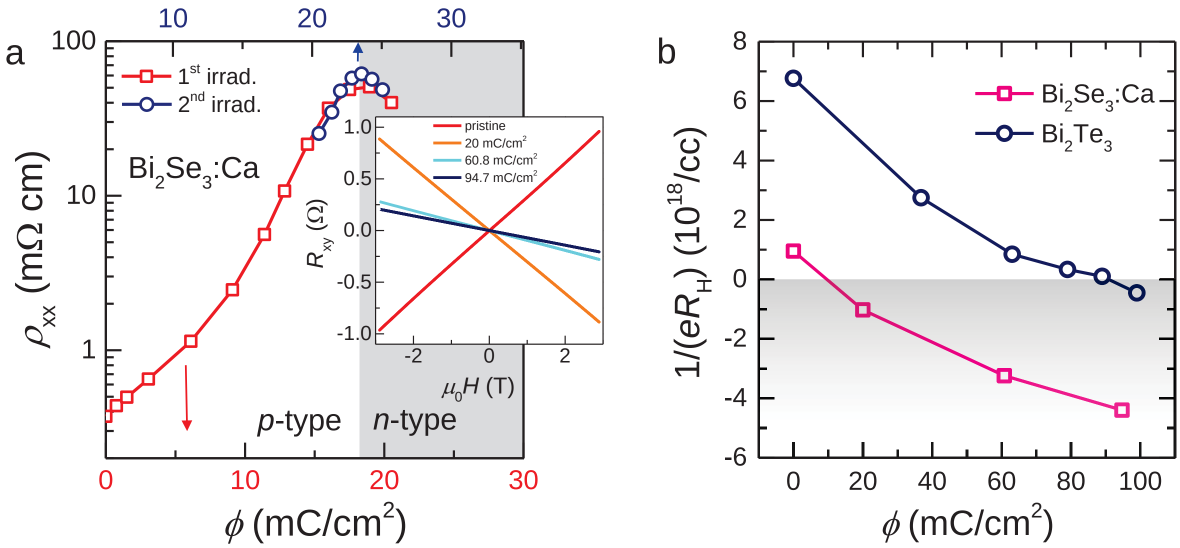

Supplementary Figure 4| Tuning through charge neutrality point (CNP): *p*- to *n*- and *n*- to *p*- conversions. (a) Longitudinal resistivity ρ_{xx} of the initially p-type Bi_2Se_3 doped with 0.09% Ca measured *in situ* at 20 K in the irradiation chamber *vs.* irradiation dose ϕ. Initial (1st) irradiation is shown as red squares. The conversion of conductance from p- to n-type tales place at $\phi_{\rm max} \cong 18$ mC/cm^2. Warming up to room temperature partially reverses the compensation process, which can be fully recovered with repeated irradiation (blue circles). The accumulated dose on the 2nd irradiation at $\rho_{\rm xx}^{\rm max}$ is $\phi_{\rm max} \cong 24$ mC/cm^2. Inset: The change of sign of the slope $\partial R_{\rm xy}/\partial H$ of Hall resistance $R_{\rm xy}$ from below $\phi_{\rm max}$ to above. (b) Inverse Hall coefficient $1/eR_{\rm H} \simeq -n_{\rm b}$ gives an estimate of net carrier density $n_{\rm b}$ as a function of dose. A quasilinear behavior of $1/eR_{\rm H}$ seen in both Bi_2Te_3 and Bi_2Se_3:Ca crystals. This universal rate of charge compensation can be used to establish the value of $\phi_{\rm max}$ at the charge neutrality point in both, bulk crystals and thin films. The key starting point is the initial carrier concentration which determines the irradiation time. Our preliminary results on thin films on SiO_2 substrates confirm this.

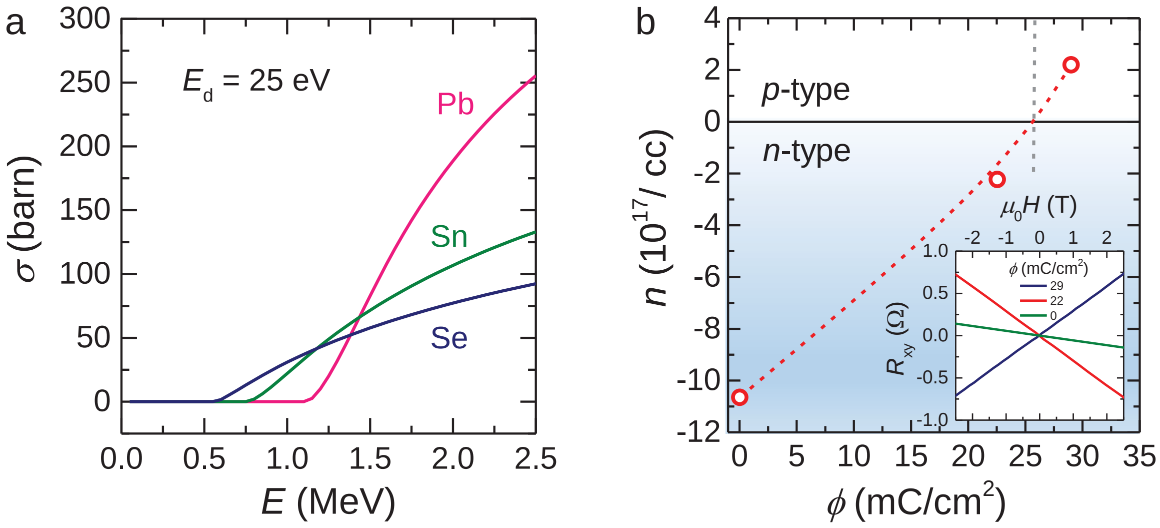

Supplementary Figure 5| Calculated crosssections σ for Frenkel pair production on Pb, Sn and Se sublattices. (a) Calculated crosssections σ for Frenkel pair production on Pb, Sn and Se sublattices as a function of electron energy E with the displacement energy $E_d = 25$ eV. (b) Carrier concentration n_b at 4 K as a function of electron dose in a topological insulator $\mathrm{Pb}_{1-x}\mathrm{Sn}_x\mathrm{Se}$ ($x = 0.3$) irradiated at 20 K. It demonstrates conversion from the initially electron (n-type) to hole (p-type) conduction at a dose $\phi \cong 25$ mC/cm^2.

Electron irradiation can be used for both p- to n- and n- to p- conversions. Since the conversion is controlled by the dominant donor or acceptor character of (mainly) vacancy partners in Frenkel pairs, it can be tuned either by electron beam energy (which chooses the dominant sublattice) or (for a given beam energy) by a suitable choice of a sublattice in another system. An example of the latter is a Pb-based topological insulator [11] $\mathrm{Pb}_{1-x}\mathrm{Sn}_x\mathrm{Se}$, where preliminary electron irradiation experiments [12] indicate the conversion of conduction from n- to p-type. This is consistent with the well known acceptor flavor of vacancies formed on the Pb sublattice [13].

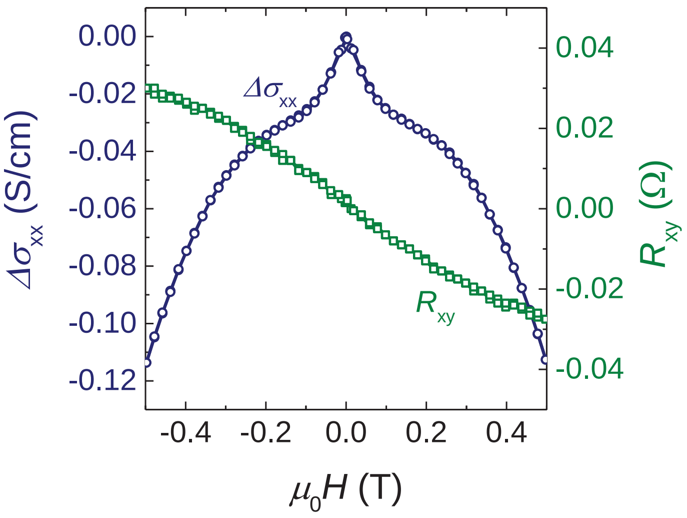

Supplementary Figure 6| Nonlinear Hall resistivity in the non-equilibrated state of irradiated Bi_2Te_3. WAL cusp in magnetoconductance of Bi_2Te_3 crystal irradiated with a low electron dose of $\phi = 90$ mC/cm^2 after dwelling for 268 hours at room temperature (for full time evolution see main Fig. 3). It is riding on a large parabolic background typical of a bulk metal or semiconductor. The Hall resistance R_{xy} shows nonlinearity which can evolve with time.

A complex temporal behavior of magnetoconductance $\sigma(H)$ is observed during room temperature annealing after electron irradiation with a relatively low dose. In this case the system is out of equilibrium and charge compensation can be locally incomplete, with two types of carriers likely to be present. In such a case Hall resistivity can be nonlinear, and this is indeed what we observe. It has been shown [14] that a net WAL feature can arise from the quantized bulk channels which may also contribute the opposing weak localization correction, WL. The background from the bulk is time-dependent; $\sigma(H)$ changes slope from positive to negative at fields beyond the field range of WAL cusp. This background is evolving in time in a manner suggestive of spatial inhomogeneity of charge compensation.

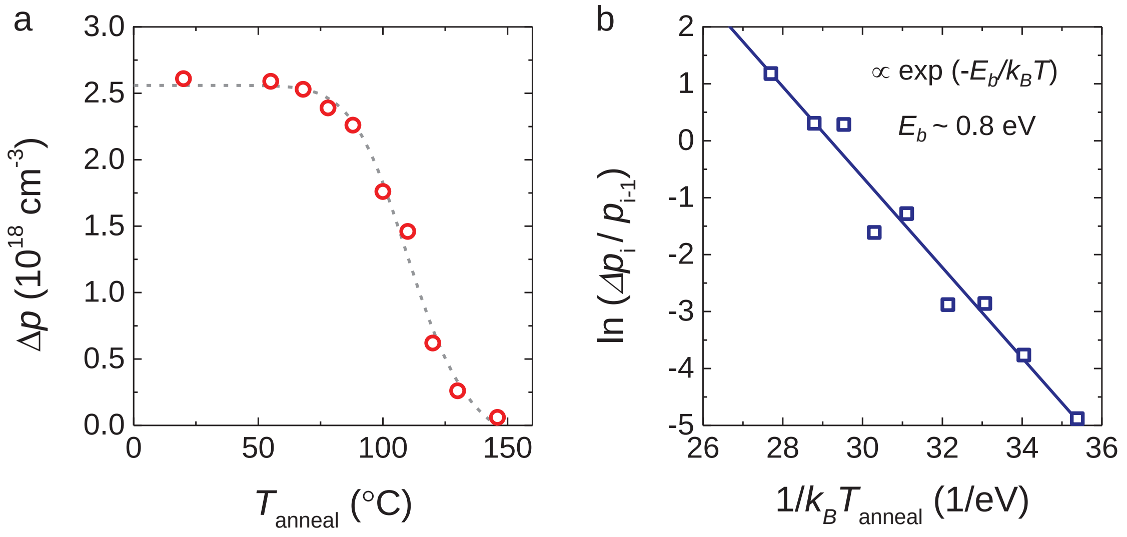

Supplementary Figure 7| Isochronal annealing experiments and energy barriers for defect migration. (a) Isochronal annealing of Bi_2Te_3 crystal irradiated to the dose of 76 mC/cm^2 in 30min intervals followed by the resistivity measurement at 4.2 K. (b) Determination of the energy barrier to defect migration from the isochronal annealing step around 100°C.

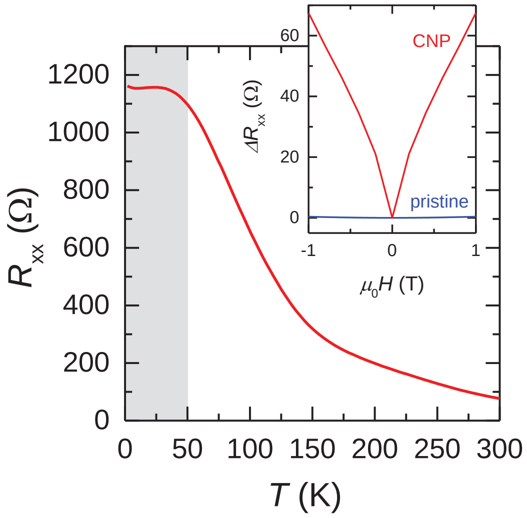

Supplementary Figure 8| Longitudinal resistance at CNP of another Bi_2Te_3 crystal. Sheet resistance R_{xx} *vs.* temperature of another Bi_2Te_3 crystal at the charge neutrality point reached through the annealing schedule shown in main Fig. 4b. Inset: Magnetoresistance at 1.9 K in a pristine Bi_2Te_3 crystal (blue) and of a crystal irradiated with electron dose of 1 C/cm^2 after annealing to CNP (red). At CNP a sharp weak antilocalization (WAL) cusp appears, which is not seen in either pristine crystals or after irradiation.

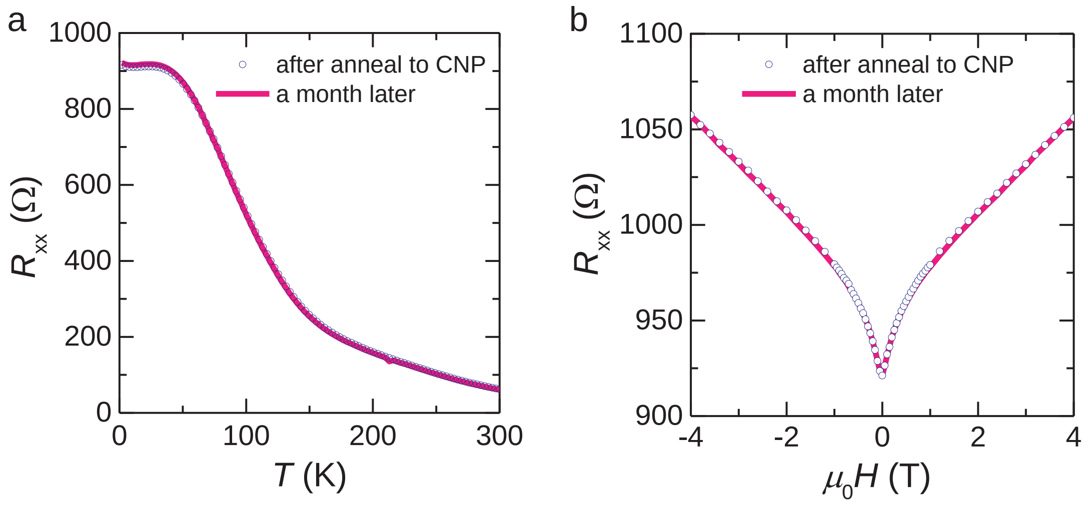

Supplementary Figure 9| Stable charge neutrality point in Bi_2Te_3 achieved through annealing. Sheet resistance R_{xx} of the Bi_2Te_3 crystal in main Fig. 4 at the charge neutrality point (a) *vs.* temperature and (b) vs. magnetic field showing the long-term temporal stability of the signal after high irradiation dose and anneal back to CNP.

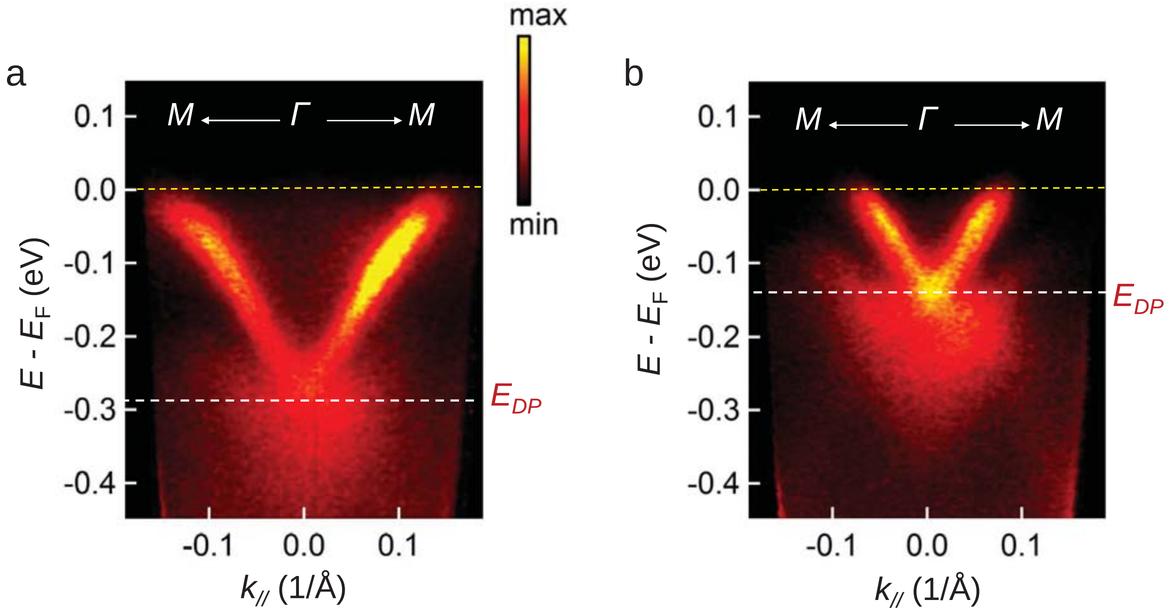

Supplementary Figure 10| ARPES of irradiated and annealed Bi_2Te_3 crystals. ARPES spectra of a Bi_2Te_3 crystal irradiated with electron dose of 1.7 C/cm^2 taken along $\Gamma - M$ direction in the Brillouin zone. (a) Before annealing the Dirac point is at $E_{DP} \sim -290(10)$ meV relative to the Fermi level E_F. (b), After 30 min anneal at 120°C Dirac point upshifts to $E_{DP} \sim -160(10)$ meV. ARPES data demonstrate that the annealing protocol that tunes the system back to CNP preserves Dirac states.

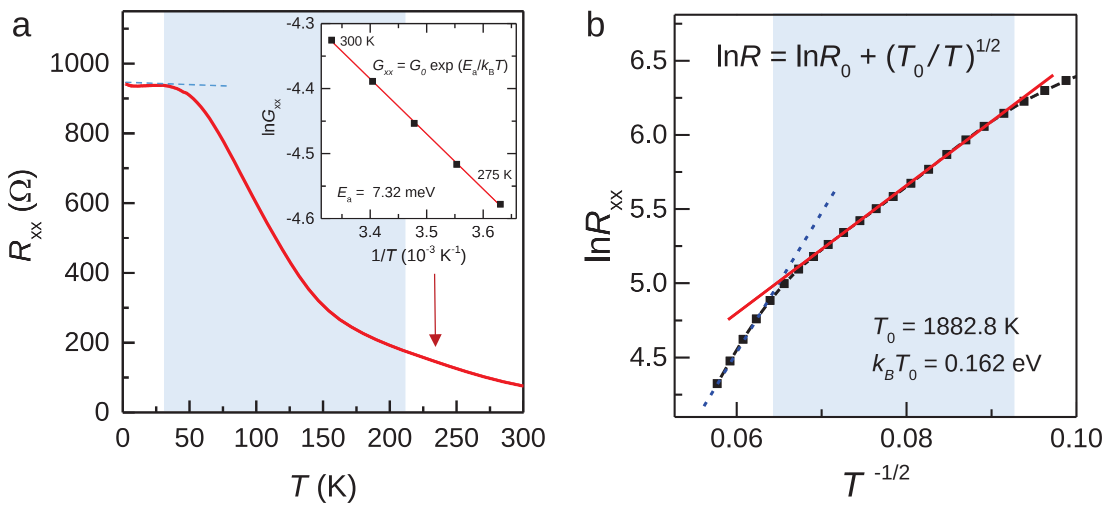

Supplementary Figure 11| Variable range hopping bulk charge transport at low carrier density. a) Sheet resistance R_{xx} *vs.* temperature of Bi_2Te_3 crystal at the charge neutrality point. Inset: A fit to a simple activation law near room temperature. (b) A fit to Efros-Shklovskii variable range hopping (VRH) law [15] law in the temperature region marked in light blue.

Supplementary Table

	k_F (Å^{-1})	n (cm^{-3})	$1/eR_H$ (cm^{-1})	μ (cm^{2}V^{-1}s^{-1})	l (nm)
**0 mC/cm²**	0.0247	5.06×10^{17}	6.8×10^{18}	11205.11	181.81
**89 mC/cm²**	0.0143	1.03×10^{17}	1.0×10^{17}	--	--
**99 mC/cm²**	0.0226	3.95×10^{17}	4.5×10^{17}	6892.39	179.53

Supplementary Table 1| Transport parameters for a pristine and irradiated Bi_2Te_3. 1st column shows irradiation doses ϕ, Fermi wavevectors k_F and the corresponding carrier densities n obtained from Shubnikov-de Haas (SdH) oscillations are in the 2nd and 3rd columns respectively. The inverse Hall coefficient which gives an estimate of carrier density at low magnetic fields is in the 4th column. The last two columns show carrier mobilities μ and mean free paths l.

Supplementary Notes

Supplementary Note 1

Tuning transport across CNP in Bi_2Se_3:Ca.

Longitudinal resistivity ρ_{xx} of the initially p-type Ca-doped Bi_2Se_3 [16, 17] measured *in situ* in the irradiation chamber kept at 20 K as a function of 2.5 MeV electron irradiation dose shows behavior identical to Bi_2Te_3 under the same irradiation conditions (see main Fig. 1e). Supplementary Fig. 4 shows two orders of magnitude resistivity increase to a maximum ρ_{xx}^{max} where the conversion from p- to n-type takes place defining charge neutrality point (CNP). Irradiation doses to achieve CNP are lower in Bi_2Se_3:Ca than in Bi_2Te_3 since the initial bulk carrier concentration is lower. As in Bi_2Te_3, for low irradiation doses $\rho_{xx}(\phi)$ is reversible upon temperature cycling to room and back to 20 K, with $\rho_{xx}(\phi)$ and ρ_{xx}^{max} traced exactly by the next irradiation cycle. Our experiments on Bi_2Te_3 were carried on more than a dozen samples cut from the same large p-type crystal and the rates of variation of carrier concentration *vs.* dose in the range 10 mC/cm^2 to 1.6 C/cm^2 were essentially the same in all samples. In all measured Bi_2Se_3:Ca crystals the rates were consistently higher than in Bi_2Te_3. We should note that access to Dirac point in Bi_2Se_3 is expected, in principle, to be less complicated than in Bi_2Te_3, since in Bi_2Se_3 DP is positioned in the bulk bandgap, while the band structure of Bi_2Te_3 (where Dirac point, DP, is nestled in the valley of the bulk valence bands) is more complex. For Bi_2Se_3 to be p-type it has to be doped, *e.g* with Ca, and Ca doping reduces carrier mobility. Our Bi_2Te_3 crystals display exceptionally high mobilities, much higher than in Bi_2Se_3:Ca which will require further improvements in the initial materials' growth. We also note that for many applications of the remarkable properties of the topological Dirac states it is not necessary to stay at DP. Indeed, to exploit the helical spin texture and strong spin polarization of the topological states one has to be above or below DP, since spins at the singular DP point are free to rotate [18].

Supplementary Note 2

Evaluating energy barriers for defect migration.

To evaluate the stability of compensating defects and to estimate energy barriers for defect migration, we performed isochronal anneals of a Bi_2Te_3 crystal irradiated with 2.5 MeV electrons at 20 K to a relatively low dose $\phi = 76$ mC/cm^2 (below type conversion) and

traced how the hole concentration p evolved relative to the initial value, positing that the incremental change Δp was proportional to the concentration of remaining defects. The initial decrease in hole concentration (here measured at 1 T) was from $3.36 \times 10^{18}\text{cm}^{-3}$ to $7.5 \times 10^{17}\text{cm}^{-3}$ obtained after cycling to room temperature (RT) and back to 4.2 K. Annealing was performed in 30 min steps each with the temperature increment of 5°C. After each annealing step, the sample was transferred into the cryostat for the measurements of resistivity and Hall effect at 4.2 K. Above $\sim$ 70°C a measurable change in carrier density (shown in Supplementary Fig. 3a) becomes apparent, and above $\sim$ 150°C the recovery is complete. For the determination of migration energy we used a standard method [4]: we calculated the increment of the carrier concentration change $\Delta p_i = p_i - p_{i-1}$ at each annealing step and normalized it to the remaining concentration of holes after the preceding step p_{i-1}, namely $\Delta p_i/p_{i-1}$. We assumed that the annealing process in each step obeys the rate equation $\text{d}p/\text{d}t = -Kp$ corresponding to exponential decay law, $p = p_\text{o}e^{-Kt}$, where K is the diffusion coefficient $K \propto e^{-E_\text{b}/kT}$ with an energy barrier E_b impeding the migration of defects. The rate of annealing in step i, $\Delta p_i/p_{i-1}$ ($\approx \text{dln}p/\text{d}t$) is proportional to K, thus the slope of $\ln(\Delta p_i/p_{i-1}) \propto \ln(e^{-E_\text{b}/kT})$ *vs.* $1/k_\text{B}T$ gives an estimate of the energy barrier controlling defect migration, $E_\text{b} \simeq 0.794$ eV (Supplementary Fig. 4b). This is a typical value for the migration energy of vacancies and over an order of magnitude above $\lesssim 0.08$ eV expected for interstitials [4]. The slow annealing process observed in a crystal irradiated to just above neutrality point in Fig. 3 of the main text is consistent with this large value of E_b; this explains why it takes hundreds of hours at RT to get back to the charge neutrality point. We note that at room temperature interstitials do not contribute since their concentration is about six orders of magnitude below that of vacancies [4].

Supplementary Note 3

Stability of surface states at CNP.

The 2D transport at CNP will have contributions from the metallic Dirac bands and from the subsurface two-dimensional electron gas (2DEG) states arising from bending of bulk bands. We note that 2DEG states become more visible in transport when the Fermi level is in the bulk bandgap. Charge transfer from the environment responsible for the band-bending would primarily be into the bulk bands nearest to the surface, if they are available. In the case of Bi_2Te_3, where Dirac point is in the valley of the bulk valence band (BVB)

[19] and where CNP crosses both Dirac and BVB, the charge transfer would be mostly into BVB and the band-bending is not a controlling factor. We should add that the subsurface 2DEG could be potentially controlled by the related ion implantation techniques, such as subsurface δ-doping or 2D single-ion doping, well developed and fine-tuned in technology of semiconductor heterostructures.

We remark that the achieved high bulk resistivity values at CNP can be particularly useful in spintronic TI-based devices. A simple estimate based on spin-torque transfer induced by spin-polarized currents through topological surfaces indicates that in a typical thin layer structure spin-torque transfer (STT) will be hugely enhanced; indeed in our irradiated TIs even at room temperature it will be only $\sim 20-30\%$ below the maximum STT expected from the spin-polarized surfaces alone. This is a large improvement over the unirradiated TIs [20] where STT is 70% below maximum owing to the significant shunting by the bulk.

Supplementary Note 4

Variable range hopping bulk charge transport at low carrier density.

Sheet resistance R_{xx} *vs.* temperature of Bi_2Te_3 crystal at the charge neutrality point (shown in main Fig. 4a) at low temperatures is temperature independent, consistent with minimum conductance $G \cong 20\ G_0$ ($G_0 = e^2/h$ is the conductance quantum) of surface conduction channels, see main text. At higher temperatures two types of activated bulk behavior are observed. With a note of caution, since the temperature range is small here, at the highest temperatures (near room temperature) a simple activation law $R_{\mathrm{xx}} \propto e^{E_{\mathrm{a}}/k_{\mathrm{B}}T}$ with a very small activation barrier $E_a \sim 7$ meV fits best (see inset in Supplementary Fig. 11a). We note that the barrier energy E_{a} appears much smaller than the bulk gap $E_{\mathrm{g}} \simeq 200-300$ meV, and well below room temperature equivalent to ~ 30 meV.

This simple activated behavior changes below ~ 220 K into variable range hopping (VRH) of the Efros-Shklovskii type: $R_{\mathrm{xx}} = R_0\exp[(T_0/T)^{1/2}]$. A fit of R_{xx} to the VRH law (Supplementary Fig. 11b) gives $E_{\mathrm{VRH}} = k_{\mathrm{B}}T_0 \sim 160$ meV equivalent to $T_0 \sim 1880$ K. Variable range hopping is a tunneling transport between separated charge puddles created by large charge fluctuations at CNP which are poorly screened, leading to unreliable estimates of the true carrier densities from Hall transport. Such behavior is expected in theory [15], where VRH temperature scale is given by $T_0 \propto 1/\xi$, and ξ is the localization length of states in the vicinity of the Fermi level measured in the units of cube root of carrier density $n^{1/3}$. Using

our value of T_0 from the fit to VRH law and carrier densities $n \sim 10^{18}$ cm^{-3}, we obtain a rough estimate [15] of $\xi \sim 0.4n^{-1/3} \approx 4$ nm. Owing to this remarkably long localization length VRH can dominate transport over a large temperature range, from $T \simeq 220$ K down to the low-temperature plateau in $R_{\rm xx}$.

Supplementary References

[1] Suh, J. *et al.* Fermi-level stabilization in the topological insulators Bi_2Se_3 and Bi_2Te_3: Origin of the surface electron gas. *Phys. Rev. B* **89**, 115307 (2014).

[2] Favre, J., Konczykowski, M., Lesueur, D. & Suski, T. Défauts dirradation dans les sels de plomb: PbTe, PbSe. *Annales de Phys.* **14**, 157–165 (1989).

[3] Rischau, C. W., Leridon, B., Fauque, B., Metayer, V. & van der Beek, C. J. Doping of Bi_2Te_3 using electron irradiation. *Phys. Rev. B* **88**, 205207 (2013).

[4] Damask, A. & Dienes, G. *Point defects in metals* (Gordon and Breach, New York, 1963).

[5] Seitz, F. On the disordering of solids by action of fast massive particles. *Discussions, Faraday Soc.* **5**, 271 (1949).

[6] Mott, N. F. The scattering of fast electrons by atomic nuclei. *Proc. R. Soc. Lond. A* **124**, 425–442 (1929).

[7] Mott, N. F. The polarisation of electrons by double scattering. *Proc. R. Soc. Lond. A* **135**, 429–458 (1932).

[8] Qi, X.-L. & Zhang, S.-C. Topological insulators and superconductors. *Rev. Mod. Phys.* **83**, 1057–1110 (2011).

[9] Murray, R., Newman, R. C. & Woodhead, J. The stability of Frenkel pairs and group V interstitials in electron-irradiated GaAs and GaP. *Semicond. Sci. Technol.* **2**, 399 – 403 (1987).

[10] Bois, P. & Beuneu, F. Defect production in Bi and dilute alloys irradiated by electrons. *J. Phys. F - Metal Phys.* **37**, 6041 (1988).

[11] Dziawa, P. *et al.* Topological crystalline insulator states in $Pb_{1-x}Sn_xSe$. *Nature Mater.* **11**, 10231027 (2012).

[12] Dybko, K., Wasik, D., Szot, M. & Konczykowski, M. Electron irradiation of topological crystalline insulator $Pb_{1-x}Sn_xSe$. *Unpublished* (2015).

[13] Abrikosov, N., Bankina, V. F., Poretskaya, L. V., Shelimova, L. & Skudnova, E. *Semiconducting II-VI, IV-VI, and V-VI compounds* (Monographs in Semiconductor Physics, p. 88, Springer, 1969).

[14] Zhang, L. *et al.* Weak localization effects as evidence for bulk quantization in Bi_2Se_3 thin films. *Phys. Rev. B* **88**, 121103(R) (2013).

[15] Skinner, B., Chen, T. & Shklovskii, B. Why is the bulk resistivity of topological insulators so small? *Phys. Rev. Lett.* **109**, 176801 (2012).

[16] Hruban, A. *et al.* Reduction of bulk carrier concentration in Bridgman - grown Bi_2Se_3 topological insulator by crystallization with Se excess and Ca doping. *J. Crystal Growth* **407**, 63 (2014).

[17] Checkelsky, J. G. *et al.* Quantum interference in macroscopic crystals of nonmetallic Bi_2Se_3. *Phys. Rev. Lett.* **103**, 246601 (2009).

[18] Zhao, L. *et al.* Singular robust room-temperature spin response from topological Dirac fermions. *Nature Mater.* **13**, 580–585 (2014).

[19] Zhang, H. *et al.* Topological insulators in Bi_2Se_3, Bi_2Te_3, and Sb_2Te_3 with a single Dirac cone on the surface. *Nature Phys.* **5**, 438–442 (2009).

[20] Mellnik, A. R. *et al.* Spin-transfer torque generated by a topological insulator. *Nature* **511**, 449 (2014).
